# Challenges and Suggestions in Management of Lung and Liver Cancer in Uzbekistan: The Second Report of the Uzbekistan–Korea Oncology Consortium

**DOI:** 10.3390/ijerph191811727

**Published:** 2022-09-17

**Authors:** Chai Hong Rim, Won Jae Lee, Bekhzood Musaev, Ten Yakov Volichevich, Ziyayev Yakhyo Pazlitdinovich, Tillysshaykhov Mirzagaleb Nigmatovich, Jae Suk Rim

**Affiliations:** 1 Department of Radiation Oncology, Korea University Ansan Hospital, Korea University, Seoul 02841, Korea; 2 Department of Healthcare Management, Gachon University, Seongnam 13120, Korea; 3 Ministry of Health of the Republic of Uzbekistan, Tashkent 100086, Uzbekistan; 4 Republican Specialized Scientific Practical-Medical Center of Oncology and Radiology, Farobiy Street 383, Tashkent 100179, Uzbekistan; 5 Department of Oral & Maxillofacial Surgery, Guro Hospital, Korea University College of Medicine, 148 Gurodong-ro, Guro 2-dong, Guro-gu, Seoul 08308, Korea

**Keywords:** lung cancer, liver cancer, hepatocellular carcinoma, Uzbekistan, South Korea

## Abstract

The health burden of cancer increases in Uzbekistan as the country develops and the life expectancy increases. Management of such a burden requires efficient screening, treatment optimization, and investigation of the causes of cancer. The Ministry of Health of Uzbekistan formed an advisory consortium, including clinical oncology and healthcare management experts from Uzbekistan and South Korea, to design a strategy for cancer management. Our consortium has analyzed six cancer types with high morbidity and mortality in Uzbekistan by classifying them into three categories (breast, cervical (gynecologic cancers), lung, liver (cancer common in men), stomach, and colorectal cancers (gastrointestinal cancers)). Lung and liver cancers are common causes of death in men after middle age—they can yield a serious health burden on the country and ruin the livelihood of families. In this review, we will analyze the oncologic literature and suggest practical recommendations for the treatment and prevention of lung and liver cancer in Uzbekistan. Data from South Korea, which has conducted nationwide screening for two decades and made progress in improving prognosis, will be discussed as a comparative control.

## 1. Introduction

In developing countries, communicable disease is the most common cause of death. As countries transition towards development, the impact of infectious diseases decreases due to the improvement of hygiene and treatment infrastructure, yielding longer lifespans and leading to non-communicable diseases accounting for the main causes of death. Two-thirds of cancers occur in people over the age of 60, and a developed socio-economic foundation is needed to establish a treatment and prevention system [1]. In sub-Saharan Africa, infectious diseases account for 50–60% of deaths, and neonatal death and malnutrition are also significant causes of mortality [2]. The proportion of cancer as a cause of death is almost negligible, because the expected lifespan does not reach the age common for cancer occurrence. In Central Asia, Southeast Asia, and North Africa, deaths from infectious diseases accounted for approximately 30% to 40% in the 1990s, and decreased to 15% to 25% in the 2010s. Alternately, the prevalence of chronic diseases and cancer significantly increased. In Western Europe, East Asia, and North America, the proportion of chronic diseases and cancer accounts for 60% or more, whereas infectious diseases account for less than 5% [2].

Uzbekistan has recently developed rapidly, and the incidence and mortality of cancer are also on the rise [3]. To respond to the increasing health burden of cancer, the Ministry of Health of Uzbekistan has formed a consortium that can provide advice on cancer treatment and prevention in Uzbekistan, including radiation oncologists, surgical oncologists, and medical informatics specialists from the Republic of Korea. Our consortium identified six major cancers with high mortality and morbidity in Uzbekistan in a previous report (breast, cervical (gynecologic cancers), lung, liver (cancer common in men), stomach, and colorectal cancers (gastrointestinal cancers)). In this report, the category including lung and liver cancer will be discussed. Target readers for this study are oncologists, healthcare providers who are not oncologists, and health policymakers.

**Figure 1:**
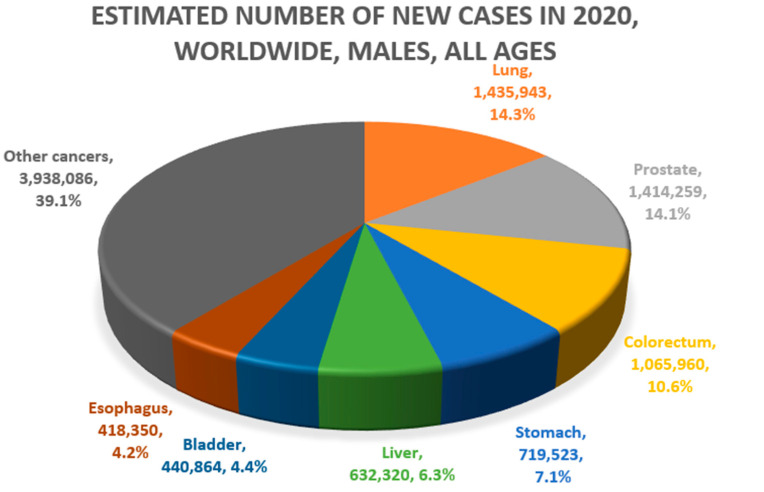

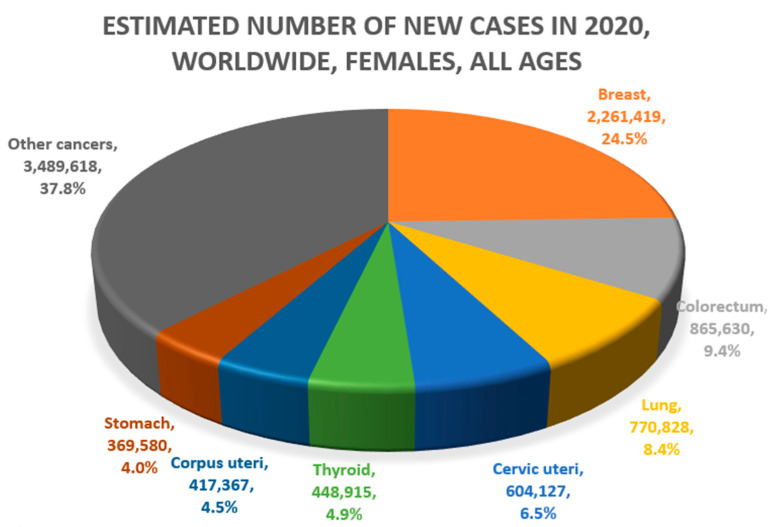
Common causes of cancer mortality in men (**above**) and women (**below**) (Data source: Global Cancer Observatory, GLOBOCAN 2020. Figures redrawn by authors) [4,5,6,7].

Lung and liver cancers are approximately 2–3 times more common in men than in women [4]. Globally, lung (21.5%) and liver cancer (10.5%) account for the first and second causes of cancer death, respectively, in men. In women, lung cancer (13.7%) is the second leading cause, and liver cancer is the sixth leading cause of death (5.7%) (Figure 1) [5]. These two diseases have been known to have relatively poor prognosis among cancers. For example, in South Korea, the 5-year survival rate for lung cancer and liver cancer was approximately 12% from 1993–1995 [1]. However, owing to the expansion of screening and advances in treatment, the 5-year survival rates for lung and liver cancer rose to 34.7% and 37.7%, respectively, from 2015–2019.

From a sociological viewpoint, lung and liver cancers are common causes of death for men after middle age; they can yield a serious health burden on countries and ruin the livelihood of families. However, academic literature on the causes and current status of liver and lung cancer in Uzbekistan is scarce. Therefore, in-depth research efforts and detailed discussion is needed. In this review, we will analyze the oncologic literature and suggest practical recommendations for the treatment and prevention of lung and liver cancer in Uzbekistan. Data from South Korea, which has conducted nationwide screening for two decades and made progress in improving prognosis, will be discussed as a comparative control.

## 2. Lung Cancer

### 2.1. Incidence and Mortality

South-central Asia (the region to which Uzbekistan belongs) has a relatively low incidence of lung cancer (age-standardized incidence of 6.6 per 100,000 people). Overall, the incidence of lung cancer corresponds with the HDI of the region, due to the longer lifespan and the fact that tobacco consumption has been first established in high-income countries [4]. The region with the highest incidence of lung cancer worldwide is Polynesia (age-standardized incidence of 37.3 per 100,000) [8], which is attributed to genetic factors or unique substances consumed rather than the smoking rate. In sub-Saharan Africa, the age-standardized incidence per 100,000 people is very low at 2.2–3.5. This is probably because the average lifespan of the country is shorter than the age at which lung cancer mainly occurs (60–70 years or older).

A characteristic feature of lung cancer epidemiology is its high mortality. As shown in Table 1, the mortality per incidence ratio in developing regions is approximately 90%. Even in the regions to which developed countries belong, the corresponding ratio is around 60%, which is higher than that of other cancers. This suggests that lung cancer is still an incurable cancer, even with advanced diagnosis and treatment techniques. The mortality per incidence was 88.2% and 64.7% in Uzbekistan and Korea, respectively. Regionally, the mortality per incidence ratios in South-central Asia, sub-Saharan African regions, Eastern Asia, Western Europe, Northern America, and Australia and New Zealand were 89.4%, 91.4–95.5%, 81.7%, 72,8%, 59.2%, and 64.3%, respectively.

### 2.2. Cause of Lung Cancer

**Figure 2:**
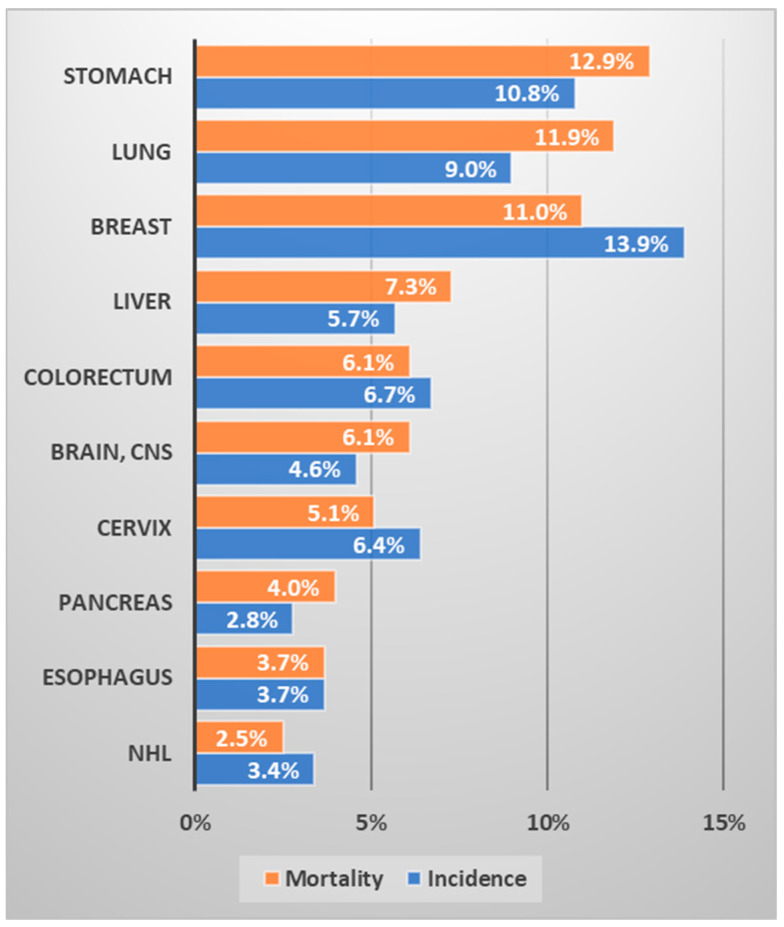
Ranking of the frequency and mortality of major cancers in Uzbekistan (data source: Cancer country profile, WHO, 2020; Global Health Observatory, WHO, 2016. Figure drawn by authors) [12,13].

It is well known that lung cancer is closely related to smoking. It has been reported that 90% of all lung cancers are related to smoking, and smokers have a 10- to 30-fold higher incidence of lung cancer than that of non-smokers [9]. The higher proportion of men who smoke than women is almost constant worldwide. Roughly speaking, about a quarter of the world’s population is smoker [10]. The smoking population in Uzbekistan was 14.4% in 2017, 26.8% among men and 1.4% among women [11]. The smoking rate in women is very low, therefore the incidence of lung cancer is expected to be low, and most lung cancer is expected to occur in men. In Uzbekistan, lung cancer ranks third (9%) in terms of prevalence and is the second leading cause of cancer death (11.9%) (Figure 2). Given that lung cancer causes a heavy health burden to society and is expected to increase, it is necessary to reduce the smoking rate, especially among men in Uzbekistan.

Although smoking is the most important risk factor for lung cancer, other factors may also contribute [11]. Chronic inflammation or infection increases the risk of lung cancer; in a meta-analysis by the International Lung Cancer Consortium, the population with chronic asthma had a 1.8 times higher incidence of lung cancer [14]. In a large Chinese study, the population with a history of tuberculosis had a 1.5–2 times higher incidence of lung cancer [15]. Radiation exposure has also been associated with lung cancer. In a pooled analysis of 13 European case-cohort studies, an increase of 16% in lung cancer incidence (95% CI: 5% to 31%) per 100 Bq/m^3^ in usual radon was reported [16]. Occupational exposure to substances such as asbestos, silica, radon, heavy metals, and polycyclic aromatic hydrocarbons is also associated with lung cancer [17]. A diet that consumes a large amount of grilled or cooked red meat over direct fire and less vegetables is also associated with lung cancer [18].

Given that Uzbekistan does not have a relatively high smoking rate, it is necessary to domestically investigate and prevent non-smoking causes of lung cancer. Although the domestic literature is lacking, hypotheses can be inferred based on studies in neighboring countries or investigating lifestyles relevant in Uzbekistan. Luqman et al. [19]. performed a case-control study including 1200 Pakistanis, finding that, in addition to smoking (odds ratio: 9.4), exposures to occupational substances, including pesticide (odds ratio: 5.1), wood dust (odds ratio: 1.9), welding fume (odds ratio: 2.5), and diesel exhaust (odds ratio 3.1) were associated with the risk of lung cancer. On the other hand, consumption of tea, coffee, and vegetables were preventive factors. In another case-control study involving 1000 Chinese women, Seow et al. [20]. reported that daily stir-fried meat cooking and its resulting fumes were significantly associated with lung cancer (odds ratio: 3.7). Uzbekistan is famous for gold mining, whereby 37.2% of national exports are gold products [21]. Of note, Kusiak et al. [22]. reported increased lung cancer mortality by 6.5% per year after 20 years of employment in Ontario gold mines. Although domestic investigation is necessary, hazardous occupational exposures or meat-cooking habits are likely to be hypothetical causes of non-smoking lung cancer in Uzbekistan.

### 2.3. Screening of Lung Cancer

The benefit of early screening for lung cancer is less significant than that for other cancers, such as breast or cervical cancers [23]. Previously, screening was attempted by performing chest radiography in Korea and other countries, but its effectiveness was unsatisfactory. Oken et al. [24] reported the effectiveness of chest X-ray screening from 1993–2001 for a population of approximately 150,000 people in the US, aged 55–74 years. However, there was no difference in lung cancer mortality compared to that of the control group without screening (risk ratio: 0.99, 95% CI, 0.87–1.22). In addition, no significant difference was reported in the results of the subgroup analysis according to smoking history. In a combined analysis of Johns Hopkins and Memorial Sloan-Kettering Cancer Center studies [25], screening with chest X-ray and sputum cytology did not significantly reduce lung cancer mortality (risk ratio: 0.88, 95% CI 0.74–1.05). However, an approximately 20% risk reduction of lung cancer death was reported in the high-risk subgroup who smoked more than 50 pack-years. Tumor markers, such as carcinoembryonic antigen (CEA), squamous cell carcinoma antigen (SCC-Ag), CYFRA 21-1, and neuron-specific enolase (NSE), do not have sufficient sensitivity and specificity for screening [26]. Therefore, screening for lung cancer was not actively recommended in Korea until around 2010. However, a large study in the US in 2012 reported that low-dose chest CT performed in a high-risk group (with a smoking history of 30 pack-years or more) could reduce lung cancer mortality by 20% [27]. Similar results were reported in a large study conducted in Europe—in a population with a smoking history of 20–30 pack years, low-dose chest CT screening reduced lung cancer mortality by 33% in women and 24% overall [27].

To summarize, the benefit of lung cancer screening is unclear compared to that of other cancers, and there are conflicting results in these studies. Most European countries do not implement national lung cancer screening, and less than 5% of the population in the US undergo annual lung cancer screening [28,29]. Meanwhile, South Korea implemented nationwide screening using low-dose chest CT for people aged 55 years or older with a smoking history of ≥30 pack-year [26]. However, to perform nationwide screening using low-dose CT, considerable consumption of medical resources is required. In addition, the high prevalence of tuberculosis and granulomatosis hinders the efficacy of nationwide screening in developing countries [23]. Therefore, screening for lung cancer in Uzbekistan is suggested selectively for high-risk groups (heavy smokers and people with a genetic history of lung cancer).

### 2.4. Treatment of Lung Cancer

Surgical resection has been the standard for stage I lung cancer, which involves a small tumor in the lung parenchyma without lymphatic metastases. For stage I disease, a 5-year survival rate of 75–80% can be obtained after successful surgery [1]. Radiosurgery can be performed, achieving comparable efficacy to surgery using the latest linear accelerator (LINAC) [30]. Stage II lung cancer is reached when the cancer has metastasized to the hilar lymph node (N1) or when it is a large tumor without lymphatic metastases (T2b-3N0). Surgical resection is commonly performed for stage II disease, and chemotherapy is considered depending on the clinical status [31]. Stage III generally refers to a disease with mediastinal lymphatic metastases. Although surgery can be considered for stage III disease, concurrent chemoradiation is the more common treatment option [31]. In South Korea, the 5-year survival rate after treatment for stage 2 and 3 (regional stage) lung cancer was reported to be 44% (2015–2019) [1]. For metastatic cancers or disease or following failed surgery or radiation therapy, chemotherapy could be the mainstay of treatment. Lung cancer is an oncological field in which the latest anticancer drugs are being rapidly developed, and various targeted therapeutics and immunotherapy drugs are being used. For example, the PD-1 inhibitor immunotherapy drug pembrolizumab has achieved a median survival of 26 months [20], and osimertinib, a drug targeting EGFR, has achieved a median survival of 38.6 months [32]. However, these therapeutic agents can only be used in patients whose targets are expressed, and the price of the therapeutic agent is very expensive.

In Uzbekistan, cancer treatment has been centered on surgical treatment. Cancer surgery is performed with certain specialties in national cancer centers and general hospitals. Minimally invasive surgery is also available at cancer centers in several regions. However, the availability of radiotherapy or chemotherapy is limited to major cancer centers. The pathology department is inefficient, with a long turnaround time and unavailability of immunohistochemistry, which could be an obstacle to the utilization of the latest immune or targeted therapies. Surgical oncologists mostly operate on breast, head and neck, colorectal, or other gastrointestinal cancers. In addition, only a few oncologists are specialized in thoracic surgery [3]. Radiation therapy plays a role in all stages of lung cancer, including radiosurgery for early disease, concurrent chemoradiation for locally advanced cancer, and palliative modality for metastases. The 2022 imPACT report described 19 external radiotherapy machines (17 Cobalt-60 and two linear accelerators (LINACs)) in Uzbekistan [3]. Considering that there were more than 230 external radiotherapy machines in South Korea in 2018, this number was grossly insufficient. Fundamentally, more LINACs need to be purchased, and more specialists need to be trained. In the short term, it is necessary to increase the treatment efficiency of Cobalt-60 through computerized planning and machine optimization [33].

### 2.5. Summary and Suggestions

The easiest way to reduce the health burden of lung cancer in Uzbekistan is to reduce the smoking rate in men. However, since Uzbekistan has a relatively low smoking rate, causes of lung cancer other than smoking should also be investigated. Screening using chest radiography or sputum cytology is ineffective. Low-dose chest CT screening can be considered for a high-risk group (heavy smokers, patients with a genetic history of lung cancer). Although anticancer drugs are being rapidly developed, the use of the latest drugs might be difficult for economic reasons. In addition, immunohistochemistry is not well utilized. There is a need to provide the best possible treatment with classical chemotherapy and radiation therapy. The number of LINACs needs to be increased, and computerized planning optimization of Cobalt-60 is required. More surgical oncologists should be specialized in thoracic surgery. In addition, they should be trained to optimize the efficacy and feasibility of lung cancer surgeries.

## 3. Liver Cancer

### 3.1. Epidemiology and Causes of Liver Cancer

Liver cancer is a generic term for cancers occurring in the liver; approximately 80% and 15% are hepatocellular carcinomas (HCC) and intrahepatic cholangiocarcinomas, respectively. Therefore, when discussing the epidemiology of liver cancer in general, HCC is mainly discussed. The incidence of liver cancer is high in East Asia and West Africa (age-standardized incidence of 17.8 and 8.4 per 100,000). The corresponding value is 6 per 100,000 in Uzbekistan, which is relatively low globally, and 14.3 in South Korea, which is relatively high. The incidence in South Central Asia is 3 per 100,000. In addition, the incidence per 100,000 in Western Europe, North America, and Australia is approximately 5–6. Mortality is very high worldwide, with the highest mortality per incidence exceeding 90% in developing regions, and 69% in Korea and North America (Table 2).

The main cause of HCC is a hepatitis virus infection. In a meta-analysis published by Shi et al., the incidence of HCC was approximately 14 times higher in the population with hepatitis B virus (HBV) and 4.6 times higher in the population with hepatitis C virus (HCV) [34]. Approximately 54% of liver cancers worldwide are associated with hepatitis B, and 31% of liver cancers are associated with hepatitis C [35]. Mongolia has the highest incidence of HCC in the world (age-standardized rate of 85.6 per 100,000), and it is estimated that more than a quarter of the population are carriers of the hepatitis B or C virus [36]. The prevalence of hepatitis B is 4–10% in East Asia, and 2–7% in Southeast Asian countries. The prevalence in Africa is as high as 8.8% (2.9–22.4%), whereas it is low (0.2–2%) in Western European countries. The prevalence in Uzbekistan is estimated to be ~7%, whereas it is 4.4% in South Korea [37]. The global prevalence of anti-HCV antibodies is approximately 1.6%. The anti-HCV prevalence in Egypt is as high as 14.7%, which is the second highest incidence of HCC in the world (34.1 age-standardized rate per 100,000). The corresponding rate in South Korea is relatively low at 0.8%; however, it is approximately 11.3% in Uzbekistan [38]. As the prevalence of both HBV and HCV in Uzbekistan is relatively high, it is estimated that the major cause of liver cancer is hepatitis virus infection. The global incidence of HCC and viral infection prevalence in the selected countries are summarized in Table 3.

The causes of HCC, other than hepatitis virus infection, include alcohol and aflatoxin consumption. Aflatoxins are usually ingested when spoiled or moldy grains are consumed. Chu et al. [39] reported a fivefold increase in the incidence of HCC in a population with a high serum concentration of aflatoxin. Regarding alcohol consumption, in a meta-analysis involving 572 studies [40], heavy drinkers who drank four or more drinks had a twofold higher risk of HCC, with a dose–response relationship. Those who drank less did not have a significantly increased risk of liver cancer. We could not find an investigation reporting the major causes of liver cancer in Uzbekistan. Therefore, it is necessary to investigate the relationship between HCC and domestic lifestyle, including the consumption of spoiled or moldy grains and heavy alcohol consumption.

### 3.2. Screening and Vaccination

As the high-risk group for HCC is well known, the use of screening for such a group has been beneficial. In South Korea, it is currently recommended to perform a liver ultrasound and serum alpha-fetoprotein testing at 6-month intervals for high-risk groups (hepatitis B and C virus carriers, cirrhosis) [41]. According to a recent study by Kwon et al. [42] that included 66,632 liver cancer patients (screening group: 10,527; no screening group: 56,105), those who underwent national screening had a higher probability of early detection (odds ratio: 1.82) and 22% reduced liver cancer mortality. In a randomized study including approximately 18,000 people in Shanghai [43], the group that underwent ultrasound and alpha-fetoprotein tests at 6-month intervals had a higher rate of detection of early liver cancer (<5 cm in size) compared to that of the control (45.3% vs. 0%). In addition, the 5-year survival rate was also significantly higher (46.4% vs. 0%) and the death rate from liver cancer was reduced by 37%. Given that the main cause of liver cancer in Uzbekistan is expected to be hepatitis virus and related cirrhosis, nationwide screening using sonography and alpha-fetoprotein testing is recommended. Although investigation is necessary to identify the main cause of HCC in Uzbekistan, the use of antiviral agents should be discussed to reduce the risk of the disease further. In a randomized study by Liaw et al. [44], lamivudine reduced the incidence of HCC in patients with hepatitis B by nearly half (hazard ratio 0.49, *p* = 0.047). However, economic considerations are necessary because a large population requires long-term drug administration.

Nationwide vaccination has effectively reduced the incidence of HBV carriers and HCC. In South Korea, a national vaccination program was implemented in 1985. In a large study including 370,285 adult Korean men, vaccinated people (vaccinated with HBsAg and anti-HBs) had a 42% reduced risk of HCC compared to that of non-vaccinated controls (unvaccinated and HBs-Ag and anti-HBs) [45]. The number of HBV carriers has also continuously decreased in recent decades [46]. In a nationwide Taiwanese study, where universal vaccine coverage started in 1986, the vaccinated cohort had a 70% reduced risk of HCC after a 20-year follow-up period [47]. Uzbekistan successfully vaccinated against HBV in 2008. The pentavalent vaccine (DTap, HepB, and Hib) was used in 2008, and its coverage exceeded 95% until recently [3]. Although hyperimmune-gamma-globulin is recommended for high-risk babies (from mothers with HBeAg+), its relatively high cost hinders its widespread use in developing countries [48]. The incidence of HCC and HBV carriers in Uzbekistan should be monitored in the long term to investigate the effectiveness of vaccination. Furthermore, the use of immunoglobulins should be discussed with economic considerations.

Social prevention against infection is important because no vaccine effective for HCV is known. HCV is transmitted through blood-related infections, such as intravenous injections, tattoos, and acupuncture. In Egypt, the prevalence of hepatitis C increased rapidly after extensive tartar emetic injection (antischistosomal treatment) between 1950 and 1980 [49]. In the United States, HCV infection was reported to be high in the hippie generation, who frequently used parenteral drug injections [50]. Therefore, it is important to investigate whether the high anti-HCV prevalence in Uzbekistan is related to lifestyle factors. In addition, the ImPACT report noted that most endoscopy centers in Uzbekistan do not have automatic cleaning devices and that they mostly use disinfectant solutions and water. Therefore, automatic washing machines are urgently required [3].

On the other hand, as a large population of Uzbekistan is engaged in agriculture, the relationship between a rural lifestyle and liver cancer should be discussed. Whether individuals are exposed to aflatoxins during the processing or consumption of grain should be investigated. The need for adequate irrigation and fungicide spraying before harvesting should be explored. After harvest, suitable crop drying methods should be adopted and moldy crops should be removed. Another method is to increase the production of crops that are not susceptible to aflatoxins [48,51,52]. A regional study in China reported that areas using pond water were five times more likely to develop HCC than areas using well water [53]. In areas where clean tap water is unavailable, at least the use of wells should be supported.

### 3.3. Treatment

Treatment of HCC includes surgical resection, liver transplantation, and radiologic interventions, such as transarterial chemoembolization (TACE) or radiofrequency ablation, chemotherapy, and radiation therapy. Surgical resection or liver transplantation has been the first option for small tumors, less than 2–3 in number, without vascular invasion [54,55]. However, HCC is often accompanied by cirrhosis at the time of diagnosis. Moreover, in countries where screening is not performed, it is commonly diagnosed as having vascular invasion or metastasis, but surgical treatment is not effective for these patients [54,56].

TACE is the most widely used palliative procedure for early- and mid-stage HCC. Radiofrequency ablation is a noninvasive treatment that can achieve results similar to those of surgery in early HCC [57]. Such interventional treatments are noninvasive and effective but require expensive equipment and skilled manpower, making them difficult to popularize in developing countries [58]. Regarding systemic chemotherapy, sorafenib was the first to demonstrate a significant survival benefit for HCC in 2008 [59]. Several drugs, including regorafenib, lenvatinib, and cabozantinib, have been introduced. Nonetheless, they did not exceed the efficacy and feasibility of sorafenib and were used as a surrogate or second-line agent of sorafenib [60]. In 2020, atezolizumab and bevacizumab combination therapy was the first to demonstrate efficacy beyond sorafenib, which has been increasingly administered recently [61]. However, these drugs cost thousands of dollars per month [62], and the magnitude of therapeutic benefit has been moderate. For example, the survival period with the use of sorafenib is increased by 2–3 months, and the treatment response rate is approximately 2% [59,63].

Radiation therapy has been commonly used for symptom relief in bone and brain metastases [64,65]. Recently, it has been actively used for palliating HCC invading major blood vessels (hepatic portal vein) in East Asian countries [66]. For HCC with vascular invasion, the median survival is less than 3 months [67]. A recent clinical series reported that radiation therapy could achieve a median survival of 11–12 months for patients with HCC with portal invasion. Furthermore, consequent surgery could achieve long-term survival in selected patients [48,50,51]. Regarding early HCCs, stereotactic radiotherapy showed similar outcomes to radiofrequency ablation in terms of local control, and is available to treat tumors near the diaphragm or surrounding blood vessels, where radiofrequency ablation is difficult to perform [68]. Although the equipment is expensive, it can be used without additional cost once installed. As radiation therapy devices have been produced in Eastern Europe, their utilization is feasible in Uzbekistan. Considering the economic aspects and treatment efficiency, radiation therapy should be actively used for HCC in Uzbekistan.

### 3.4. Summary and Suggestions

The prevalence of hepatitis virus infection in Uzbekistan is relatively high. A rural lifestyle may be related to the development of HCC. A domestic investigation is needed to discover the common cause of HCC in Uzbekistan. Administration of the hepatitis B vaccine is well performed and should be maintained. Screening using ultrasound and alpha-fetoprotein tests for high-risk groups (hepatitis virus carriers and patients with liver cirrhosis) is recommended. Radiation therapy should be actively used to improve the prognosis of liver cancer. Efforts are necessary to establish radiological intervention equipment and training specialists. Economic and medical discussions on the necessity of antiviral agents are needed.

## 4. General Summary and Recommendations

### 4.1. Summary and Recommendations

As HDI increases in Uzbekistan and life expectancy increases, the prevalence of lung and liver cancers also increases. Although these two cancers are intractable, their prognosis should be improved through active prevention strategies and therapeutic optimization. Domestic research is needed to identify the causes of lung and liver cancer.

The primary objective of reducing the incidence of lung cancer in Uzbekistan is to promote smoking cessation, especially among men (strongly recommended). Early screening using chest X-rays did not reduce lung cancer mortality. Low-dose chest CT could reduce lung cancer mortality by approximately 20% in the high-risk group (over 55 years of age, >30 pack-years), and selective screening is recommended for high-risk groups (recommended). The infrastructure for radiation therapy should be expanded and actively performed. In addition, specialized thoracic surgical oncologists should be trained (recommended). Domestic research is needed to determine whether there are any causes of lung cancer other than smoking. Socioeconomic discussions are necessary for utilization of the latest systemic agents.

Hepatitis virus infection is the main cause of HCC and its prevalence is relatively high in Uzbekistan. National screening using liver sonography and alpha-fetoprotein is strongly recommended for established high-risk groups (hepatitis virus carriers or cirrhosis patients) (strongly recommended). A nationwide hepatitis B vaccination program should be maintained, and social sources of HCV infection should be identified (e.g., inadequate endoscopy cleaning, etc.) (strongly recommended). Radiologic intervention infrastructure and specialists should be trained. In addition, external radiation therapy should be actively utilized (recommended). Causes of liver cancer other than the hepatitis virus (e.g., exposure to aflatoxin by spoiled grain) should be investigated. Socioeconomic and medical discussions are necessary for the utilization of antiviral and systemic agents for HCC.

### 4.2. Recommendations for Further Research

Cancer research in Uzbekistan has been rudimentary, few physicians are participating in prospective or translational research. Most healthcare providers have difficulty to conduct research due to heavy clinical load, inconsistent data, and lack of motivation. The government and hospitals should create a social system and research funds where healthcare providers can build academic careers. General hospitals are recommended to collect clinical information through standardized interview methods. Given that most centers still used paper-based records, electronic records systems should be communized [3]. In Supplementary Table S1, we summarized recommended subjects and methodology for further research based on our review.

## Figures and Tables

**Table 1:** Brief global statics of lung cancer, including Uzbekistan and South Korea.

Region or Country	Uzbekistan	South Korea	South-Central Asia	Sub-Saharan African Regions	East Asia	Western Europe	Northern America	Australia and New Zealand
Incidence	8.5	25.5	6.6	2.2–3.5	34.4	32.7	32.6	25.2
Mortality	7.5	16.5	5.9	2.1–3.2	28.1	23.8	19.3	16.2
Mortality per incidence	88.2%	64.7%	89.4%	91.4–95.5%	81.7%	72.8%	59.2%	64.3%

All values are age-standardized rates per 100,000 population. Data source: incidence and mortality are from Global Cancer Observatory, GLOBOCAN 2020 [4,5,6,7].

**Table 2:** Brief global statistics of liver cancer including Uzbekistan and South Korea.

Region or Country	Uzbekistan	South Korea	South-Central Asia	Sub-Saharan African Regions	East Asia	Western Europe	Northern America	Australia and New Zealand
Incidence	6	14.3	3	4.6–8.4	17.8	5.4	6.8	6.1
Mortality	5.6	9.9	2.8	4.3–8.1	16.1	4.5	4.7	41
Mortality per incidence	93.3%	69.2%	93.3%	93.5–96.4%	90.4%	83.3%	69.1%	67.2%

All values are age-standardized rates per 100,000 population. Data source: incidence and mortality are from GLOBOCAN 2020 [4,5,6].

**Table 3:** Epidemiology of viral hepatitis and hepatocellular carcinoma.

	Uzbekistan	Iran	South Korea	China	Mongolia	Indonesia	Egypt	Cameroon	Nigeria	Germany	Denmark	USA
Chronic HBV infection (%)	7.0%	1.0%	4.4%	5.5%	9.1%	1.9%	1.7%	12.2%	9.8%	0.7%	0.9%	0.3%
Anti HCV prevalence (%)	11.3%	0.5%	0.8%	1.3%	10.8%	0.8%	14.7%	11.6%	8.4%	0.4%	0.7%	1.3%
HCC incidence (age-standardized, per 100,000)	6	6.8	14.3	18.2	85.6	7.9	34.1	6.3	5.2	4.3	4.9	6.9

HBV, hepatitis B virus; HCV, hepatitis C virus; HCC, hepatocellular carcinoma. Data source: Chronic HBV infection rate from Schweitzer et al. [37]. Lancet 2015; anti-HCV prevalence rate from Gower et al. [38]. J Hepatol 2014; incidence of HCC from GLOBOCAN 2020 [4,5,6].

## Supplementary Materials

The following supporting information can be downloaded at: https://www.mdpi.com/article/10.3390/ijerph191811727/s1, Table S1. Suggested subjects for further research.
