# Supplementary material for: Challenges and Suggestions in Management of Lung and Liver Cancer in Uzbekistan: The Second Report of the Uzbekistan–Korea Oncology Consortium
Source: Int J Environ Res Public Health. 2022 Sep 17;19(18):11727. doi: 10.3390/ijerph191811727 (PMC9517504; doi:10.3390/ijerph191811727)
Supplement: Supplementary file 1 [file ijerph-19-11727-s001.zip › ijerph-1894971-supplementary.pdf]

Supplement Table S1. Suggested subjects for further research

Disease	Subject	Topic	Recommended Methodology
Lung cancer	Prevention and etiology	Cause of lung cancer in Uzbekistan	Cohort or case-control study<br>Questionairre-based study
		Non-smoking cancer cause in Uzbekistan (e.g. occupational exposure: wood dust, disel exshaust, mining dust, cooking fume)	
		Investigating efficacy and feasibility of chest CT screening	Governance study<br>Systematic review
	Treatment	Thoracic surgeon training program	Delphi study<br>Systematic review
		Standardized protocol of pathologic specimen preparation and reporting (including immunohistochemistry)	
		Optimizing Cobalt-60 plan for lung cancer	Case-series study<br>Translational research
		Investigating effect and feasibility of novel systemic treatment	Governance research
Liver Cancer	Prevention and etiology	Cause of liver cancer in Uzbekistan	Cohort or case-control study<br>Questionairre-based study
		Nationwide effect of hepatitis B virus vaccination	
		Route of hepatitis C infectoin in Uzbekistan	Case-control study<br>Questionairre-based study
		Investigating efficacy and feasibility of liver cancer screening (e.g. sonography or alphafeto protein measurement)	Governance study<br>Systematic review
	Treatment	Investigating effect and feasibility of systemic treatment (e.g. sorafenib, anti-viral agents)	Governance research<br>Systematic review
		Investigating effect and feasibility intervention radiology	
		Use of external radiotherapy on locally advanced HCC	Case-series study<br>Translational research
